# Supplementary material for: Conceptualising alcohol consumption in relation to long-term health conditions: Exploring risk in interviewee accounts of drinking and taking medications
Source: PLoS One. 2019 Nov 7;14(11):e0224706. doi: 10.1371/journal.pone.0224706 (PMC6837440; doi:10.1371/journal.pone.0224706)
Supplement: S1 Appendix — (DOCX) [file pone.0224706.s001.docx]

S1 Appendix: types of prescribed medication taken by interviewees

| **Type of medicine** | **Examples mentioned** |
| --- | --- |
| opioids | morphine, fentanyl, codeine, oxycodene, methadone substitution therapy |
| Tranquilisers and hypnotics | diazepam and zopiclone |
| antidepressants | amitryptiline, mirtazapine, sertraline, venlafaxine |
| antimetabolites | methotrexate |
| antipsychotics | respidon |
| anticonvulsants | tegretol (carbamazepine), pregabalin |
| non-opioid, non-steroidal analgesics | nefopam, paracetamol |
| anti-arrhythmics | amiodarone |
| anti-platelets | clopidogrel, ticagrelor |
| anticoagulants | warfarin |
| statins | simvastatin, atorvastatin |
| nitrates | isosorbide mononitrate, glyceryl trinitrate |
| potassium channel activators | nicorandil |
| ACE inhibitors | rampiril, Lisinopril |
| beta-adrenergic receptor blocking agents | bisoprolol |
| angiotensin receptor blockers | iosartan, candesartan |
| alpha-1 adrenergic blockers | doxazosin |
| calcium channel blockers | amlodipine, verapamil |
| diuretics | furosemide, bendroflumethiazide |
| nonsteroidal anti-inflammatories | naproxen, aspirin, ibuprofen |
| proton pump inhibitors and H2 (histamine-2) blockers | omeprazole, lansoprazole, ranitidine |
| laxatives | bisacodyl, senna, laxido, ispaghula husk, movicol |
| antimalarials (for statin linked leg cramps) | quinine sulfate |
| anti-emetics | piperazines |
| bronchodilators and corticosteroids | ventolin, seretidem, salbutamol, symbicort inhalers |
| antihistamines and corticosteroids | cetririzine, fluticasone furoate, ioratadine |
| alpha blockers for urination symptoms related to prostate problems | tamsulosin, vesomni |
| thyroid medicines | thyroxine, levothyroxine |
| diabetes medicines | empagliflozin, saxagliptin |
| mitochondrial disease medication |  |
| autoimmune disease infusions | rituximab |
| corticosteroids for polymyalgia rheumatica |  |
| antibiotics for pericardial mastitis |  |
